# Integrated Analysis of Fatty Acids and Phenolic Compounds in *Agriophyllum squarrosum* (L.) Moq.: A Promising Desert Crop for Functional Foods and Sustainable Health

**DOI:** 10.3390/biom16070950

**Published:** 2026-06-26

**Authors:** Yuliya Genievskaya, Magzhan Almukhamed, Pengshan Zhao, Saule Abugalieva, Yerlan Turuspekov, Alibek Zatybekov

**Affiliations:** 1 Laboratory of Molecular Genetics, Institute of Plant Biology and Biotechnology, Almaty 050040, Kazakhstan; julia.genievskaya@gmail.com (Y.G.); magzanalmuhamed905@gmail.com (M.A.); absaule17@gmail.com (S.A.); yerlant@yahoo.com (Y.T.); 2 State Key Laboratory of Ecological Safety and Sustainable Development in Arid Lands, Northwest Institute of Eco-Environment and Resources, Chinese Academy of Sciences, Lanzhou 730000, China; zhaopengshan@lzb.ac.cn

**Keywords:** desert pseudocereal, ecotype differentiation, metabolic profiling, functional food potential

## Abstract

*Agriophyllum squarrosum* (L.) Moq. is a desert-adapted pseudocereal that has recently attracted attention as a climate-resilient crop and source of valuable phytochemicals and nutritionally relevant metabolites. Despite their ecological and nutritional importance, comprehensive studies combining lipid and phenolic profiles across natural populations remain limited. In the present study, five populations of *A. squarrosum* from ecologically contrasting regions of Kazakhstan were analyzed to evaluate biochemical diversity and potential for functional food applications. Total lipid content was determined using near-infrared spectroscopy, fatty acid composition was assessed by GC-MS, and phenolic compounds were quantified by HPLC. Multivariate approaches, including PCA, MANOVA, PLS analysis, correlation networks, and TOPSIS ranking, were applied to evaluate population differentiation and relationships between biochemical traits and environmental conditions. Total lipid content in seeds ranged from 7.71% to 15.40%, linoleic acid represented 50.20–57.67% of total fatty acids, and oleic acid ranged from 24.80% to 40.10%. Isorhamnetin was the dominant phenolic compound in leaves, with concentrations between 0.24 and 0.65 mg/g. Populations from Aktobe showed higher lipid and oleic acid contents, whereas Almaty populations accumulated greater flavonoid levels, including isorhamnetin, quercetin, and kaempferol. These findings reveal substantial metabolic differentiation among populations and suggest possible associations with ecological conditions. The observed accumulation of unsaturated fatty acids and phenolic compounds, including isorhamnetin, quercetin, and kaempferol, identifies promising germplasm resources for future studies on functional food development and biological activity evaluation. The results further support the potential utilization of *A. squarrosum* in sustainable agriculture in arid regions.

## 1. Introduction

Global climate change, desertification, and increasing pressure on agricultural production have intensified interest in stress-adapted crops that can maintain productivity and nutritional quality under harsh environmental conditions. In this context, underutilized pseudocereals and desert plants are increasingly recognized as valuable genetic and biochemical resources for sustainable agriculture and future food security [1,2,3,4,5,6]. Among them, *Agriophyllum squarrosum* (L.) Moq. (sand rice) has attracted growing attention due to its exceptional tolerance to drought, high temperatures, nutrient-poor soils, and sand burial, as well as its potential nutritional value [5,7,8,9,10,11].

*A. squarrosum* is an annual psammophytic species widely distributed across the arid and semi-arid regions of Central Asia, Mongolia, Siberia, and northern China [7,8,9]. Besides its ecological importance in dune stabilization and desert restoration [12,13,14], sand rice has historically been used as a food and forage resource in Central Asia [8,15]. Kazakhstan represents one of the major centers of its natural distribution, where previous studies have documented substantial genetic diversity and adaptive potential among natural populations, highlighting the value of this species for conservation and future crop improvement programs [9,16].

Recent studies have demonstrated that *A. squarrosum* seeds are rich in proteins, dietary fiber, minerals, and unsaturated fatty acids, particularly nutritionally important polyunsaturated fatty acids (PUFAs) [5,17,18,19]. Previous reports have also suggested that sand rice may possess protein and dietary fiber contents comparable to those reported for quinoa under certain environmental conditions [16,18]. In addition, metabolomic investigations revealed the presence of diverse phenolic compounds, including quercetin, kaempferol, isorhamnetin derivatives, and phenolic acids [20,21,22].

Phenolic compounds play important roles in plant adaptation to environmental stress and are frequently used as biochemical indicators of ecological responses and local adaptation [22,23,24,25,26,27,28,29,30,31,32,33]. Previous studies have shown that environmental gradients influence the accumulation of phenolic metabolites in *A. squarrosum*, suggesting a close relationship between metabolite composition and habitat conditions [34,35]. Among the reported metabolites, isorhamnetin appears to be one of the dominant phenolic constituents of sand rice [34]. Several flavonoids identified in *A. squarrosum*, including isorhamnetin, quercetin, and kaempferol, have previously been reported to have been reported to possess diverse biological activities, including antioxidant and anti-inflammatory effects [36,37,38]. However, most available evidence regarding these biological activities originates from studies of isolated compounds or extracts from other plant species, whereas direct bioactivity assessments of *A. squarrosum* populations remain limited. Therefore, characterization of metabolite composition represents an important first step for identifying populations that may be prioritized for future biological and functional evaluations.

Despite increasing interest in the nutritional and metabolic properties of *A. squarrosum*, comprehensive studies simultaneously evaluating seed fatty acid composition and leaf phenolic profiles across natural populations remain limited. Most previous investigations have focused either on ecological adaptation or on targeted analyses of individual metabolite groups [34,35]. Consequently, the extent of biochemical differentiation among natural populations and its potential relevance for germplasm utilization remain insufficiently understood.

We hypothesized that natural populations of *A. squarrosum* originating from contrasting climatic regions of Kazakhstan would exhibit significant differentiation in fatty acid and phenolic profiles, reflecting local environmental adaptations and providing valuable biochemical diversity for future use. Therefore, the aim of this study was to conduct an integrated biochemical characterization of five natural populations of *A. squarrosum* by analyzing seed lipid and fatty acid composition together with leaf phenolic profiles. Furthermore, multivariate statistical approaches were used to evaluate population differentiation and explore associations between biochemical traits and environmental factors. Because fatty acids and phenolic compounds are frequently used as indicators of nutritional quality and potential biological functionality, their characterization may help identify populations suitable for future bioactivity-oriented investigations. The obtained results provide new insights into the biochemical diversity of this desert pseudocereal and may support future germplasm evaluation, breeding efforts, and subsequent studies addressing biological activities in arid environments.

## 2. Materials and Methods

### 2.1. Plant Material and Sampling

The five natural populations of *A. squarrosum* were sampled in 2025 across three ecologically distinct regions of Kazakhstan: Aktobe (AK01, AK02), Almaty (AL01, AL02), and Kyzylorda (KO01). These regions represent western, southeastern, and southern arid zones, respectively. These sandy terrains represent the typical natural habitats for this species, providing the characteristic substrate necessary for its development. Geographic coordinates of sampling sites were recorded using a handheld GPS device Garmin eTrex 10 (Garmin Corporation, Taiwan, China). Spatial distribution of populations was visualized using Google Earth tools (Google Earth Pro, version 7.3.6, Google LLC, Mountain View, CA, USA).

All sampled accessions represented naturally occurring wild populations growing in their native habitats. No experimental cultivation or transplantation was involved. Field sampling was conducted between August and September 2025 during the reproductive stage, when seeds had reached physiological maturity, and leaves were fully developed. Detailed geographic coordinates, altitude, collection dates, and habitat characteristics are provided in Table 1. All populations were located in sandy desert habitats characterized by low organic matter content and sparse vegetation cover, which represent typical ecological conditions for *A. squarrosum*.

Ten individual plants were randomly sampled from each population and treated as independent biological replicates. To minimize the probability of sampling closely related individuals, plants were collected at intervals of approximately 10–20 m. Seeds and leaves from each plant were processed separately, and all analytical measurements were performed in triplicate and treated as technical replicates. Leaf samples were air-dried under ambient conditions and stored in paper bags containing silica gel at room temperature (22–25 °C) for less than two months prior to analysis. The moisture content of dried material was maintained below 10%. Only one population was sampled in the Kyzylorda region because no additional naturally occurring populations that met the predefined sampling criteria were available during the survey period. Climatic parameters were summarized to describe environmental conditions, including mean annual temperature (MAT), monthly temperature patterns, mean annual precipitation (MAP), and seasonal precipitation dynamics. Monthly temperature (°C) and precipitation (mm) data for five sampling sites were obtained from a publicly available climatic database (https://www.kazhydromet.kz/ru/, accessed on 12 May 2026).

### 2.2. Total Lipid Content Analysis

Total lipid content in *A. squarrosum* seeds was determined using an Infralum FT-12 NIR analyzer (Lumex, St. Petersburg, Russia) in accordance with ISO 12099:2017 [39] with calibration provided by the manufacturer. Cleaned seed samples (50 g) of each population were scanned in triplicate over the 800–2500 nm range, and spectral data were processed using SpectralLUM/Pro ver. 4.01.393 software with PLS regression. The final total lipid content was as a percentage of seed dry weight (DW).

### 2.3. Fatty Acid Analysis

Fatty acid composition was determined by gas chromatography–mass spectrometry (GC-MS). All measurements were conducted in three replications. Methanol, chloroform, sodium methoxide, n-hexane, and a 37-component fatty acid methyl ester (FAME) standard mixture were obtained from Sigma-Aldrich (St. Louis, MO, USA). Fatty acid methyl esters were prepared using a two-step transesterification procedure. Initially, 10 g of seeds from each sample were ground and treated with 1 M sodium methoxide in methanol at 50 °C for 15 min to convert glycerides. This was followed by acid-catalyzed methylation using methanolic HCl to ensure complete conversion of free fatty acids and polar lipids. The resulting FAMEs were extracted with n-hexane, washed with saturated NaCl solution, dried over anhydrous sodium sulfate, and appropriately diluted to ensure detector response within the linear range [40].

GC-MS analysis was performed using a Shimadzu Nexis GC2030 system (Shimadzu Corporation, Kyoto, Japan) coupled with a GCMS-QP2020 NX mass spectrometer (Shimadzu Corporation, Kyoto, Japan) and equipped with an SH-Rt-2560 capillary column (100 m × 0.25 mm × 0.20 µm) (Shimadzu Corporation, Kyoto, Japan). Helium was used as the carrier gas at a constant flow rate of 1.02 mL/min. The oven temperature program was set as follows: an initial temperature of 40 °C held for 2 min, followed by a ramp of 4 °C/min to 240 °C, with a final hold to ensure complete elution of long-chain fatty acids, resulting in a total run time of 65 min. Mass spectrometric detection was performed in scan mode (*m*/*z* 45–500), with selective ion monitoring of major fatty acids to enhance sensitivity.

Fatty acids were identified by comparing retention times and mass spectra with those of a certified 37-component FAME standard mixture and the NIST spectral library. Quantification was performed using the internal standard method (Figure S1).

Compound identification was accepted only when both retention times and mass spectral patterns matched those of the certified FAME standards and NIST library entries. Relative retention times are provided in Supplementary Table S1. Quantification was based on peak-area normalization and expressed as a percentage of the total identified fatty acids (Table S1).

### 2.4. Phenolic Compound Analysis

A total of 5 g of dried leaf samples of *A. squarrosum* was finely ground and extracted with 80% methanol containing 0.1% formic acid at a solvent-to-solid ratio of 20:1 (*v*/*w*). The extraction procedure was adopted from a previously published protocol [41] and provided extracts of sufficient quality for subsequent HPLC analysis. The combined extracts were subsequently concentrated under reduced pressure using a rotary evaporator (RV 3V, IKA, Staufen, Germany) at 40 °C to remove methanol and prevent thermal degradation of thermolabile phenolic compounds. The concentrated residue was re-dissolved in 15 mL of methanol and centrifuged at 4000 rpm for 10 min. Prior to HPLC analysis, the extracts were filtered through 0.45 µm membrane filters [41].

High-performance liquid chromatography (HPLC) analysis was performed using a Shimadzu LC-40D XR HPLC system equipped with an SPD-M40 diode array detector (DAD), CTO-40C column oven, SIL-40C XR autosampler, and DGU-405 degasser (Shimadzu Corporation, Kyoto, Japan). Chromatographic separation was performed on a Waters Symmetry C18 reversed-phase column (250 mm × 4.6 mm, 5 µm particle size) (Waters Corporation, Milford, OH, USA). The mobile phase consisted of (A) deionized water containing 0.4% orthophosphoric acid (49685, Sigma-Aldrich) and (B) HPLC-grade methanol (34860, Sigma-Aldrich). Separation was performed under isocratic conditions using 53% solvent A and 47% solvent B at a flow rate of 1.0 mL/min. The column temperature was maintained at 30 °C, and the total run time was 30 min. Injection volume ranged from 10 to 20 µL.

Identification of phenolic compounds was performed by comparing retention times and UV spectra (200–400 nm) with authentic analytical standards obtained from Sigma-Aldrich: isorhamnetin (17794), kaempferol (60010), quercetin (Q4951), coumarin (C4261), rhamnetin (17799), syringic acid (S6881), and vanillic acid (94770). Detection wavelengths were optimized individually for each compound: isorhamnetin at 355 nm, kaempferol at 366 nm, quercetin at 370 nm, coumarin at 277 nm, rhamnetin at 370 nm, tricin at 353 nm, syringic acid at 275 nm, and vanillic acid at 261 nm. External calibration curves were prepared for each analytical standard using at least five concentration levels. Calibration linearity was assessed using linear regression, and only calibration models with coefficients of determination (R^2^) exceeding 0.99 were accepted for quantification. Analytical repeatability was evaluated by triplicate measurements of each extract, and the relative standard deviations were below 5% for the major detected compounds (Table S1).

### 2.5. Statistical and Multivariate Analysis

Statistical analyses were performed to evaluate variation and relationships among seed biochemical traits. Basic descriptive statistics, including minimum, maximum, mean, standard deviation (SD), variance, and coefficient of variation (CV), were calculated for all measured traits. The Principal Component Analysis (PCA) was performed separately for two groups of traits: fatty acids with total lipids and phenolic compounds. Pairwise relationships among all traits were evaluated using Pearson’s correlation coefficients (*p* < 0.05), and correlation matrices were visualized. Additionally, correlation-based network analysis was conducted to examine the structure of relationships among biochemical traits. The network was constructed using significant pairwise correlations (r > 0.7, *p* < 0.05). Phenotypic relationships among individual samples were further explored using a UPGMA clustering approach [42] based on distance matrices derived from integrated traits in PAST4 [43]. Multivariate analysis of variance (MANOVA) was performed to test for significant differences among populations based on studied biochemical traits (separately for fatty acids with total lipids, phenolic compounds, and the combined dataset). Multivariate significance was assessed using Pillai’s Trace statistic [44].

A partial least squares (PLS) regression analysis was performed to evaluate the relationships between climatic variables and biochemical traits. Mean annual precipitation (MAP) and mean annual temperature (MAT) were used as predictor variables in two separate, independent PLS tests: one evaluating total lipids and fatty acids, and the other evaluating phenolic compounds as response variables. All variables were mean-centered and unit variance scaled prior to analysis. Model performance was evaluated using R2X, R2Y, and Q2 statistics. The PLS model was fitted with two components and validated using leave-one-out cross-validation (LOOCV) to assess the strengths of climate–trait associations.

The technique for order of preference by similarity to ideal solution (TOPSIS) analysis was used to evaluate the overall performance of the samples across all traits. All traits were treated as benefit criteria, except the content of linoleic acid, palmitic acid, stearic acid, arachidic acid, behenic acid, and coumarin, which were considered cost criteria. The decision matrix was normalized using min–max normalization to remove scale effects. Entropy weighting, calculated according to Shannon entropy theory [45], was applied to assign objective weights based on the information content and variability of each trait. TOPSIS was performed by defining ideal and anti-ideal solutions, followed by calculating the Euclidean distances of each accession from these reference points. A relative closeness coefficient was computed as the final TOPSIS score. Populations were ranked accordingly, with higher scores indicating superior overall performance.

All analyses were carried out using R software (R Core Team, 2023), and multivariate analyses and data visualization were performed using standard R packages, including corrplot, RColorBrewer, igraph, scales, ggplot2, virids, hrbrthemes, ggrepel, dplyr, pls, tidyr, and vegan [46].

## 3. Results

### 3.1. Environmental Characteristics of Sampling Sites

**Figure 1:**
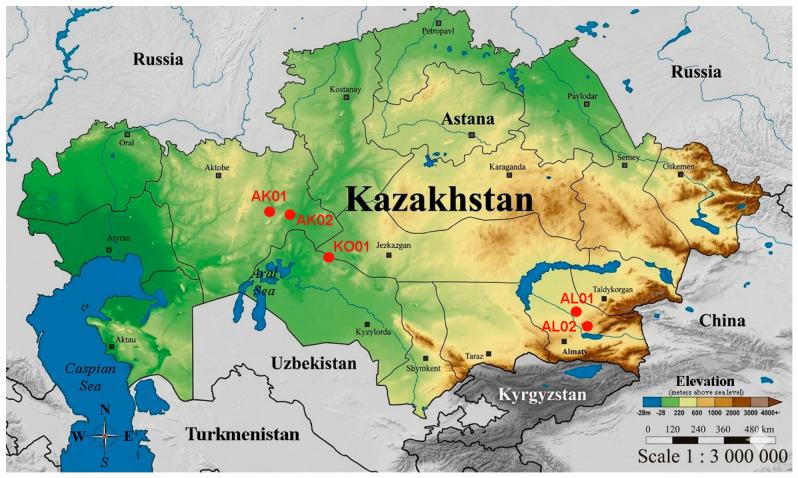
The geographical locations of the collected *A. squarrosum* populations (red dots) across the Aktobe (AK01 and AK02), Almaty (AL01 and AL02), and Kyzylorda (KO01) regions.

The spatial distribution of five *A. squarrosum* populations collected across three regions of Kazakhstan is shown in Figure 1. Two populations were sampled in Aktobe (AK01, AK02), two in Almaty (AL01, AL02), and one in the Kyzylorda region (KO01).

**Figure 2:**
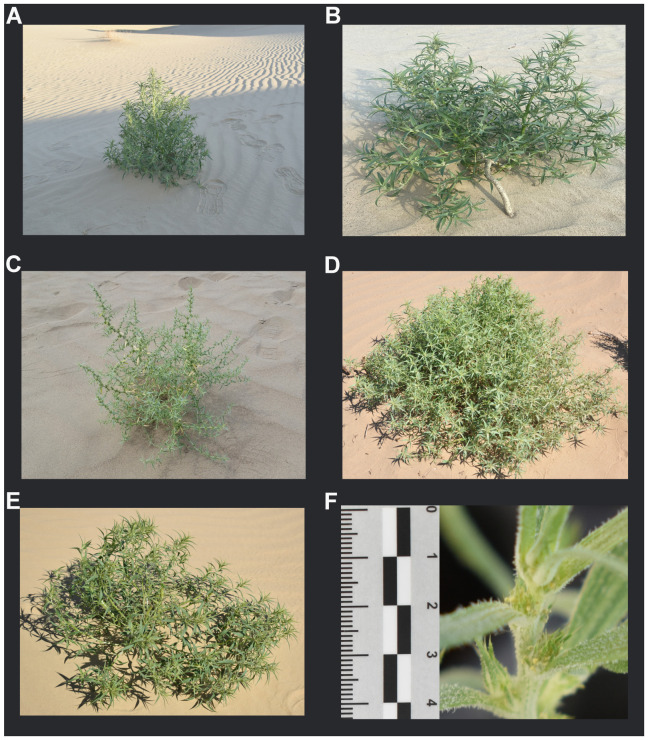
Morphological diversity and growth habit of *A. squarrosum* populations collected from the deserts of Kazakhstan. (**A**) AK01, Aktobe region; (**B**) AK02, Aktobe region; (**C**) AL01, Almaty region; (**D**) AL02, Almaty region; (**E**) KO01, Kyzylorda region; (**F**) Close-up view of vegetative and reproductive structures of *A. squarrosum*.

The sampling design captures a west–south–southeast gradient encompassing geographically and ecologically distinct environments. The Aktobe populations are located in western Kazakhstan, while the Kyzylorda represents southern arid conditions, and the Almaty populations correspond to southeastern environments with more heterogeneous landscapes (Figure 2).

**Figure 3:**
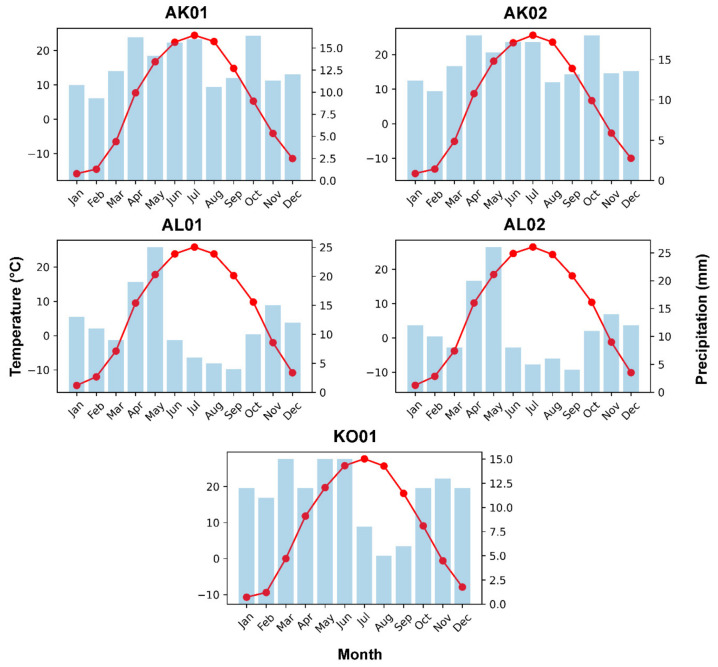
Monthly patterns of mean air temperature (red line) and precipitation (blue bars) across five study locations in 2025.

All sites exhibit a clear seasonal temperature trend, with minimum values observed in January–February and maximum values in July (Figure 3).

Winter temperatures at all locations are below 0 °C, reaching a minimum of −18.17 °C at AK01, while summer temperatures, on average, exceed +20 °C, peaking at +29.99 °C at KO01.

Precipitation shows pronounced variability across months. In AK01 and AK02, precipitation ranges between 9.3 and 18 mm, with higher values in spring (April–May) and autumn (October). AL01 and AL02 exhibit higher precipitation peaks than other sites, reaching 26 mm in May, whereas the summer months (July–September) show reduced values, often below 10 mm. KO01 exhibits moderate precipitation variability, with higher values in spring (March–May) and autumn (October–November), and lower values in summer. Across all locations, precipitation was generally lowest in late summer (August–September) and increased again toward autumn. The temporal alignment of precipitation peaks differs slightly among sites but consistently occurs outside peak summer temperatures.

### 3.2. Total Lipid Content and Fatty Acid Composition

**Figure 4:**
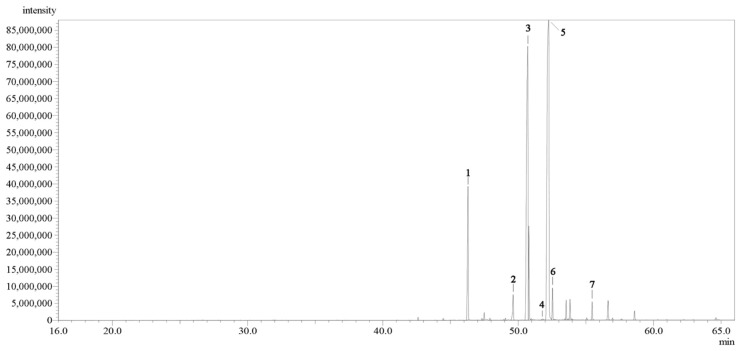
Representative GC-MS total ion chromatogram (TIC) of an *A. squarrosum* seed sample (AK01, individual No. 7), showing the major identified fatty acid. 1—Methyl palmitate (palmitic acid), 2—Methyl stearate (stearic acid), 3—Methyl oleate (oleic acid), 4—Methyl linolelaidate (α-linolenic acid), 5—Methyl linoleate (linoleic acid), 6—Methyl arachisate (arachidic acid), 7—Methyl behenate (behenic acid).

**Figure 5:**
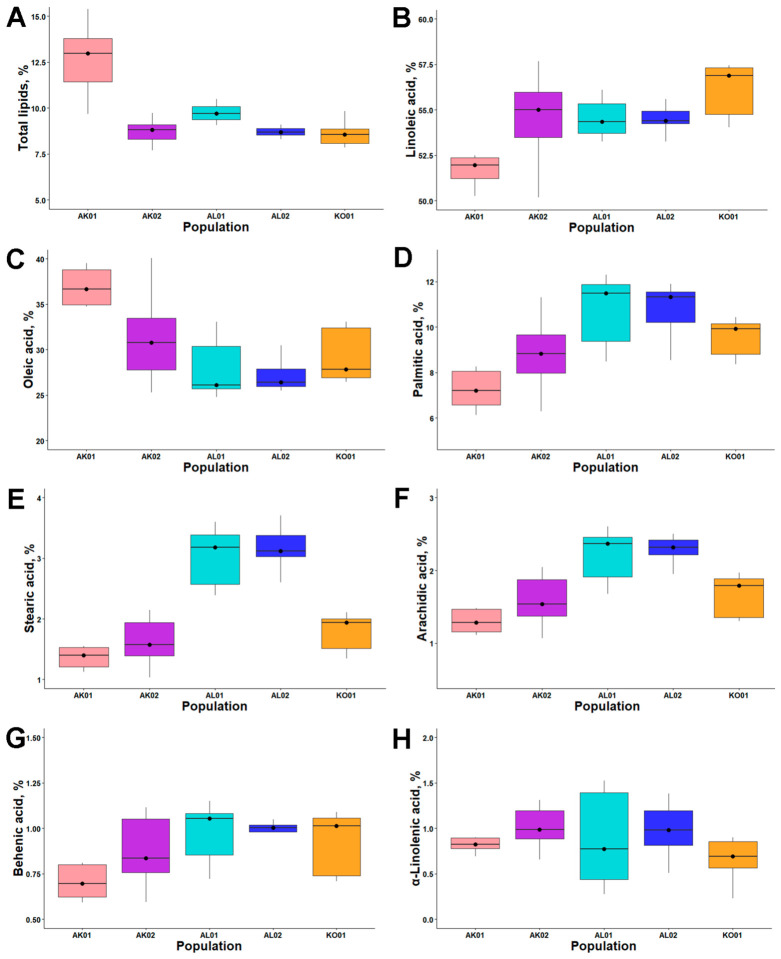
Box plots showing the variation in total lipids and seven fatty acids concentrations across the five studied *A. squarrosum* populations. (**A**)—Total lipid content; (**B**)—Linoleic acid; (**C**)—Oleic acid; (**D**)—Palmitic acid; (**E**)—Stearic acid; (**F**)—Arachidic acid; (**G**)—Behenic acid; (**H**)—α-Linolenic acid.

The total lipid content and fatty acid composition analysis revealed distinct accumulation patterns among populations (Figure 4 and Figure 5). Population-level mean values and standard deviations for all fatty acid traits are presented in Table S2.

Total lipid content (Figure 5A) ranges from 8% to 14% among populations. AK01 demonstrates an elevated median total lipid level, while the remaining populations exhibit lower median values within a narrower range, with the minimum observed in KO01. Linoleic acid is the dominant fatty acid across all populations, with concentrations ranging from 50.2% to 57.7%. KO01 exhibits the highest median values, while AK01 shows the lowest (Figure 5B). The interquartile ranges indicate moderate variability within populations. Oleic acid displays a wider distribution, ranging from 24.8% to 40.1% (Figure 5C). AK01 is characterized by higher medians, whereas AL01 and AL02 exhibit lower median values with relatively narrow spreads. Palmitic acid ranges from 6.1% to 12.3%, with higher median values observed in AL01 and AL02 (Figure 5D). Stearic acid ranges from 1.03% to 3.71%, with AL01 and AL02 showing elevated median levels relative to other populations (Figure 5E). Arachidic acid concentrations range from 1.07% to 2.6%, with AL01 and AL02 exhibiting higher values (Figure 5F). Behenic acid shows a narrower range (0.59–1.15%) (Figure 5G). α-Linolenic acid ranges from 0.23% to 1.52%, with wider dispersion observed in AL01 (Figure 5H).

**Figure 6:**
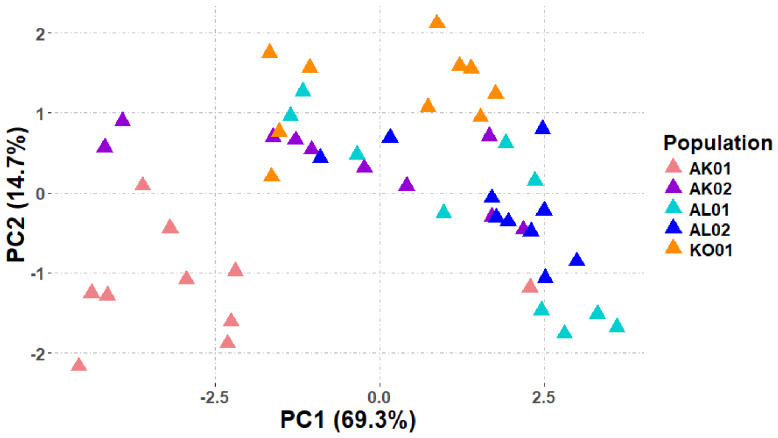
Principal Component Analysis (PCA) showing the population structure and clustering of *A. squarrosum* based on total lipids and fatty acids profiles.

The PCA plot was constructed based on the total lipids and fatty acids profiles. The first principal component (PC1) explains 69.3% of the total variance, while the second principal component (PC2) accounts for 14.7%, resulting in a cumulative variance of 84.0% (Figure 6).

Samples are clearly separated along PC1, with additional dispersion along PC2. Generally, they formed two primary clusters. The first one is positioned on the left side of the plot and includes the majority of the AK01 population, as well as two samples from the AK02 population. The remaining populations and samples of AK01 and AK02 formed a separate cluster on the right. Samples in the second cluster were mostly intermixed.

### 3.3. Phenolic Compounds Analysis

**Figure 7:**
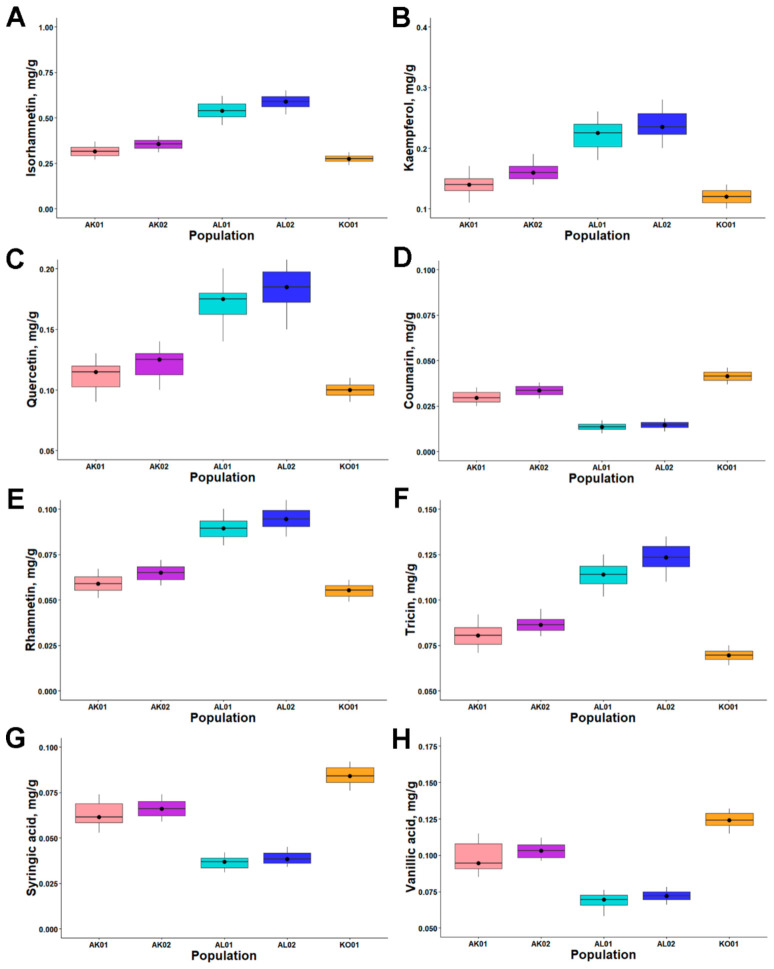
Box plots showing variation in phenolic compounds across the studied *A. squarrosum* populations. (**A**)—Isorhamnetin; (**B**)—Kaempferol; (**C**)—Quercetin; (**D**)—Coumarin; (**E**)—Rhamnetin; (**F**)—Tricin; (**G**)—Syringic acid; (**H**)—Vanillic acid.

The distribution patterns of phenolic compounds vary across the studied *A. squarrosum* populations (Figure 7). Population-level mean concentrations and standard deviations of phenolic compounds are presented in Table S2.

The highest concentrations were observed for isorhamnetin, followed by kaempferol and quercetin. Isorhamnetin concentrations range from 0.24 to 0.65 mg/g, with higher values observed in AL01 and AL02 and lower values in KO01 (Figure 7A). Kaempferol ranges from 0.1 to 0.28 mg/g, with median values increasing from AK01 to AL02 and being lower in KO01 (Figure 7B). Quercetin shows a range of 0.09–0.22 mg/g, with AL01 and AL02 exhibiting higher values than AK01, AK02, and KO01 (Figure 7C). Coumarin displays low absolute concentrations, ranging from 0.01 to 0.05 mg/g, with KO01 showing the highest values and AL01 and AL02 the lowest (Figure 7D). Rhamnetin varies from 0.05 to 0.11 mg/g, with higher values observed in AL01 and AL02 (Figure 7E). Tricin ranges between 0.06 and 0.14 mg/g, with AL02 exhibiting the highest median values (Figure 7F). Syringic acid concentrations range from 0.03 to 0.09 mg/g, with KO01 showing higher values and AL01 and AL02 lower values (Figure 7G). Vanillic acid ranges from 0.06 to 0.13 mg/g, with KO01 exhibiting the highest median value (Figure 7H).

**Figure 8:**
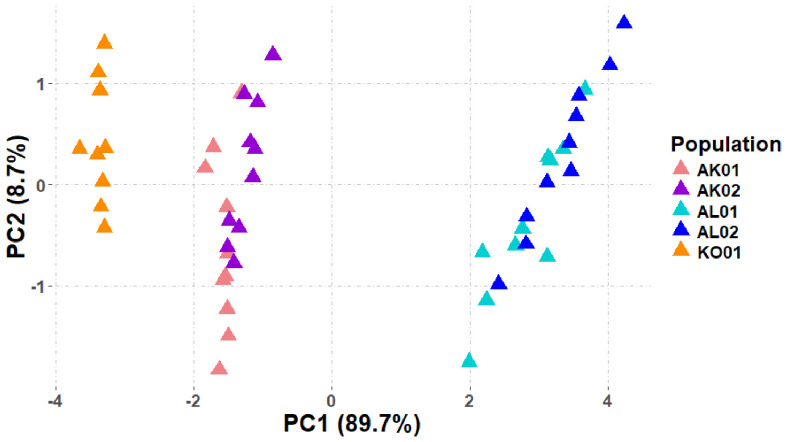
Principal Component Analysis (PCA) showing the population structure and clustering of *A. squarrosum* based on phenolic compounds.

As with the total lipid and fatty acid profiles, PCA was performed on the phenolic compounds dataset. The distribution of samples shows clear separation along PC1 (89.7%) of the total variance, while the second principal component (PC2) accounts for 8.7%, resulting in a cumulative variance of 98.4% (Figure 8).

As a result, three distinct clusters were formed based on the population’s geographical origins. KO01 formed a separate cluster on the left. Samples from AK01 and AK02 were grouped together in the second cluster at the center. Finally, populations AL01 and AL02 were also grouped together on the right.

### 3.4. Integrated Correlation Structure and Phenotypic Relationships

**Figure 9:**
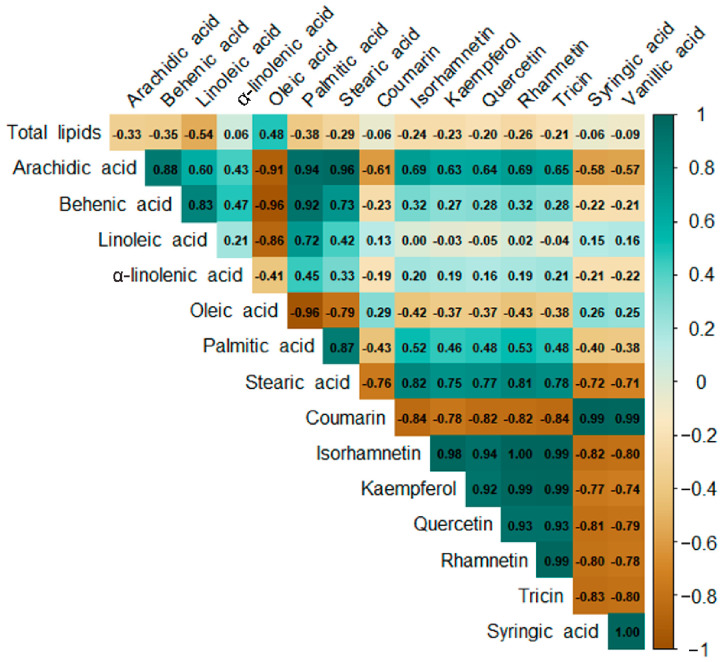
Correlation matrix heatmap illustrating the Pearson correlation between seed fatty acids and leaf phenolic compounds in *A. squarrosum* populations.

The correlation matrix presents Pearson correlation coefficients among total lipid content, fatty acids, and phenolic compounds in *A. squarrosum* populations (Figure 9).

Strong positive correlations are observed among flavonoids, including isorhamnetin, kaempferol, quercetin, and rhamnetin, with coefficients ranging from 0.92 to 1.00. Tricin also shows positive correlations with these compounds (r = 0.93–0.99). In contrast, coumarin exhibits strong negative correlations with flavonoids (r = −0.78 to −0.84) and strong positive correlations with syringic and vanillic acids (r = 0.99). Syringic and vanillic acids are strongly positively correlated (r = 1.00) and show negative associations with most flavonoids (r = −0.74 to −0.83).

Total lipid content is negatively correlated with most phenolic compounds, with coefficients ranging from −0.06 to −0.26. Among fatty acids, arachidic acid shows strong positive correlations with palmitic acid (r = 0.94), stearic acid (r = 0.96), and behenic acid (r = 0.88). Behenic acid is positively correlated with palmitic acid (r = 0.92) and linoleic acid (r = 0.83). Linoleic acid exhibits strong negative correlations with oleic acid (r = −0.86) and moderate positive correlations with palmitic acid (r = 0.72) and stearic acid (r = 0.42). α-linolenic acid shows moderate positive correlations with palmitic (r = 0.45) and stearic acids (r = 0.33). Oleic acid is strongly negatively correlated with palmitic acid (r = −0.96) and stearic acid (r = −0.79).

Because the present study included only five natural populations, the observed correlations should be interpreted as exploratory statistical associations rather than evidence of direct biological or causal relationships. Additional studies involving larger population sets and experimental validation will be required to confirm these patterns.

**Figure 10:**
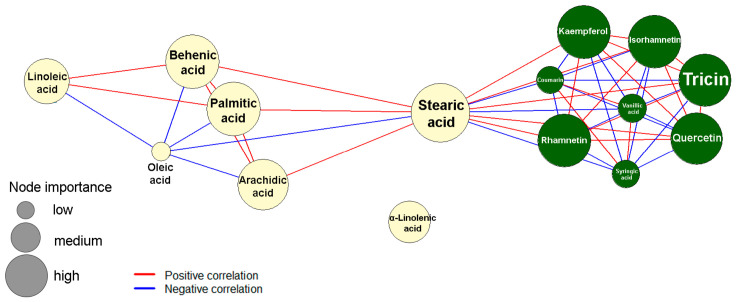
Correlation network analysis of seed fatty acids and leaf phenolic compounds across *A. squarrosum* populations.

The correlation network reveals a clear structural division into two primary, interconnected modules based on positive (red lines) and negative (blue lines) correlations, alongside an isolated element (Figure 10).

The right module is composed entirely of flavonoids (tricin, isorhamnetin, kaempferol, quercetin, rhamnetin), phenolics (vanillic and syringic acids), and coumarin. Within this dense cluster, nodes are extensively interconnected by positive and negative correlations. Tricin, isorhamnetin, and kaempferol display high node importances, as indicated by their larger node sizes. The left module consists of 5 fatty acids (linoleic, behenic, palmitic, oleic, and arachidic acids). These components show positive and negative internal correlations. Stearic acid was positioned between two groups, acting as a central hub node of high importance that links the other fatty acids and phenolic compounds. Finally, α-linolenic acid remains completely isolated with no network connections.

**Figure 11:**
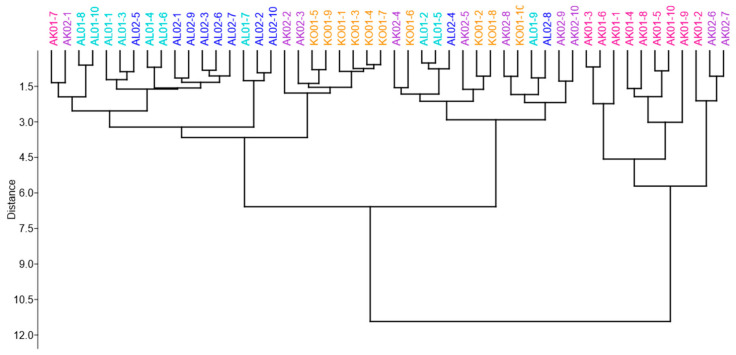
UPGMA tree based on integrated seed fatty acid and leaf phenolic profiles of *A. squarrosum*. The colors represent the different populations as follows: pink—Aktobe1, purple—Aktobe2, light blue—Almaty1, dark blue—Almaty2, and orange—Kyzylorda1.

The UPGMA dendrogram illustrates hierarchical clustering based on integrated biochemical components across *A. squarrosum* samples (Figure 11).

Samples were grouped into several distinct clusters, with separation occurring at multiple hierarchical levels. The major bifurcation divides the dataset into two primary clusters at a distance of 11. Within these main clusters, further sub-clustering is observed at lower distance levels, generally between 6 and 3. Individual samples within populations tend to group together at shorter distances, typically below 3, forming compact subclusters.

The AK01 population exhibits distinct isolation, with nine of its ten individuals grouping with two samples from AK02 on the far right of the dendrogram, forming a highly specific primary cluster that diverges from all other populations at the root.

The remaining population group is within the large primary cluster on the left, which splits into two mixed sub-clusters. The first sub-branch aggregates the AL01 and AL02 samples together, while also including a distinct group of six KO01 individuals. The second sub-branch primarily focuses on the remaining KO01 population and AK02 individuals.

### 3.5. Statistical Validation of Population Differentiation

Table 2 summarizes variation in 17 seed biochemical traits among all collected populations of *A. squarrosum*. Raw data are presented in Table S3, whereas population-level mean values and standard deviations for all measured traits are presented in Table S2. The total lipid content ranges from 7.71% to 15.40% (mean value 9.69 ± 1.80). Linoleic acid is the most abundant fatty acid (mean 54.35 ± 1.98). Stearic and α-linolenic acids exhibit relatively high variability (CV > 35%).

Among phenolic compounds, coumarin has the highest variability (CV = 42.59%), followed by isorhamnetin and syringic acid. Mean concentrations range from 0.03 mg/g (coumarin) to 0.42 mg/g (isorhamnetin). The highest mean concentration was observed for isorhamnetin (0.42 ± 0.13 mg/g), while the lowest was for coumarin (0.03 ± 0.01 mg/g).

MANOVA was conducted to assess the significance of biochemical differentiation among the five populations studied. The analysis, based on Pillai’s Trace statistic, revealed highly significant differences among populations across three trait categories—all traits, total lipids and fatty acids, and phenolic compounds (Table 3).

Significant multivariate variation among populations was observed for all three trait categories (*p* < 9.5 × 10^−16^). Pillai’s Trace indicated the strongest population differentiation when all traits were analyzed jointly, followed by phenolic compounds and total lipids with fatty acids.

**Figure 12:**
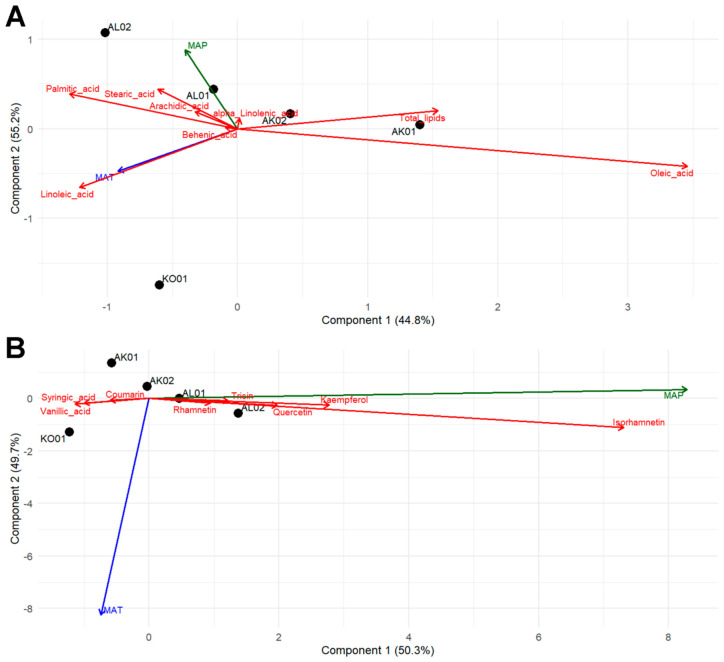
PLS biplot showing the relationship between climatic variables and seed fatty acids (**A**) and leaf phenolic compounds (**B**) of *A. squarrosum*. Black circles represent populations; red arrows indicate individual biochemical variables. Green and blue arrows signify the environmental parameters MAP (Mean Annual Precipitation) and MAT (Mean Annual Temperature), respectively.

PLS biplots reveal distinct biochemical profiles among the evaluated *A. squarrosum* populations based on total lipids, fatty acids, and phenolic compounds (Figure 12).

In the PLS biplot of total lipids and fatty acid profiles (Figure 12A), Component 1 and Component 2 represented the primary and secondary latent variables, explaining 44.8% and 55.2% of the total covariance between the measured variables, respectively. A strong positive correlation is observed between the AK01 and AK02 populations and high concentrations of oleic acid and total lipids along Component 1. Conversely, the AL01 and AL02 populations associate closely with saturated fatty acids, including palmitic, stearic, and arachidic acids, while the KO01 population aligns primarily with linoleic acid and the MAT parameter.

In the phenolic profile biplot (Figure 12B), Component 1 (50.3%) and Component 2 (49.7%) separate the populations based on distinct metabolite associations. The MAP vector demonstrates a strong positive orientation along Component 1, grouping closely with kaempferol, quercetin, tricin, rhamnetin, and isorhamnetin. Populations AK01, AK02, and AL01 partition toward the negative quadrant of Component 1, characterized by higher relative abundances of coumarin, syringic acid, and vanillic acid. The MAT vector strongly influences Component 2 in the negative direction, projecting away from both the phenolic compounds and most sampled populations.

**Figure 13:**
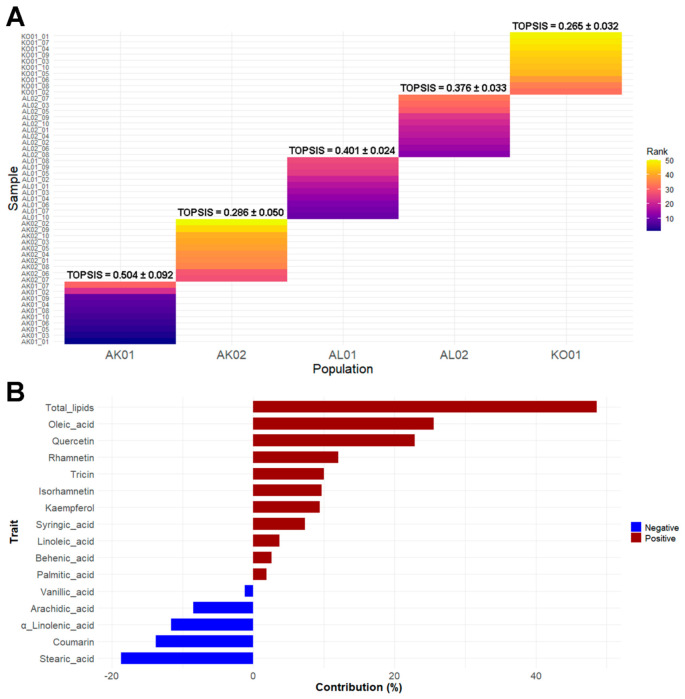
Multi-traits TOPSIS ranking based on integrated seed fatty acid composition and leaf phenolic compound profiles of *A. squarrosum*. (**A**) Heatmap stratification of individual samples and populations ordered by TOPSIS scores. The mean TOPSIS scores for each population are shown. (**B**) The percentage contribution of total lipids, fatty acids, and phenolic compounds to the composite TOPSIS score.

The observed relationships between climatic variables and biochemical traits represent statistical associations identified within the studied population set. Given the limited number of populations included in the analysis, these associations should be interpreted cautiously and do not necessarily imply causal environmental effects. Comprehensive multi-criteria evaluation using TOPSIS establishes a clear ranking of the A. squarrosum populations based on their integrated traits across total lipids, fatty acids, and phenolic compounds (Figure 13).

The population-level TOPSIS scores follow a descending order from AK01 to AL01, AL02, AK02, and KO01, which corresponds to a parallel shift in sample rankings from top positions to lower tiers (Figure 13A). Full TOPSIS scores per sample are provided in Table S4. AK01 showed the best overall biochemical profile, followed by AL01 and AL02, highlighting their potential for nutritional and functional applications.

Analysis of individual trait contributions elucidates the biochemical drivers of these performance scores (Figure 13B). Total lipids are the largest positive contributor to the ranking, accounting for 48.6%, followed by oleic acid (25.5%) and quercetin (22.8%). Other positive phenotypic drivers include rhamnetin, tricin, isorhamnetin, kaempferol, and syringic acid. Conversely, stearic acid has the strongest negative impact on the overall TOPSIS score (−18.7%), with coumarin (−13.8%), α-linolenic acid (−11.6%), and arachidic acid (−8.5%) also being major negative contributors. In total, the contents of total lipids, oleic acid, quercetin, and stearic acids were major contributors to the TOPSIS score.

## 4. Discussion

### 4.1. Biochemical Diversity and Ecological Differentiation of A. squarrosum Populations

The results of this study revealed substantial biochemical variation among *A. squarrosum* populations collected in three regions of Kazakhstan. The observed population differentiation was associated with geographic and climatic variation among sampling sites [15]. However, because the present study evaluated only five natural populations, the detected relationships should be interpreted as indicative patterns rather than direct evidence of environmental causation.

Clear regional differences were observed in both lipid metabolism and phenolic compound accumulation. Aktobe populations (AK01, AK02) exhibited elevated total lipid content and higher oleic acid proportions, whereas Almaty populations (AL01, AL02) accumulated greater levels of saturated fatty acids and flavonoids. The mean total lipid content (9.69 ± 1.80%) was consistent with values previously reported for the species (10.6% dry matter) [17], confirming the nutritional richness of *A. squarrosum* seeds (Table 1). Linoleic acid predominated across all populations, while substantial variation in oleic acid content suggests environmentally regulated shifts in fatty acid desaturation. Because seed oil unsaturation is strongly influenced by temperature [47], the higher levels of oleic acid in Aktobe populations likely reflect adaptation to cooler climatic conditions.

Compared with fatty acids, phenolic compounds displayed even stronger population-level differentiation, highlighting the high environmental sensitivity of secondary metabolism. Populations from the Almaty region accumulated significantly higher concentrations of isorhamnetin, quercetin, kaempferol, rhamnetin, and tricin. The strong association between precipitation and the accumulation of phenolic compounds observed in the PLS analysis supports the role of these metabolites in protection against abiotic stress (Figure 12B). Flavonoids such as quercetin and kaempferol are well known for their antioxidant activity and for mitigating oxidative damage caused by drought and UV exposure [48].

In contrast, the southern population KO01 exhibited elevated levels of coumarin, syringic acid, and vanillic acid, suggesting a different metabolic strategy associated with adaptation to extreme heat and aridity. This pattern corresponded with the orientation of the MAT vector in the phenolic PLS biplot (Figure 12B). These compounds are commonly linked to stress signaling and antimicrobial defense [49]. The observed biochemical variability appears to reflect adaptive metabolic plasticity shaped by long-term climatic selection pressures. These findings emphasize the importance of *A. squarrosum* populations from Kazakhstan as valuable reservoirs of adaptive traits for dryland agriculture and future domestication programs.

### 4.2. Nutritional Quality and Fatty Acid Composition for Food Use

The fatty acid composition of *A. squarrosum* confirms its strong potential as a nutritionally valuable pseudocereal suitable for dryland agriculture. Similar to quinoa and buckwheat, sand rice is a gluten-free seed enriched in unsaturated fatty acids, supporting its potential for functional food development [18]. The total lipid content observed in this study ranged from 7.71% to 15.40%, with an average of 9.69 ± 1.80% (Table 2), which is consistent with, or slightly higher than, previously reported data for the species [17].

Linoleic acid was the dominant fatty acid across all populations, accounting for more than half of the total fatty acid pool and confirming *A. squarrosum* as a rich source of essential polyunsaturated fatty acids (PUFAs). However, substantial interpopulation variation was observed in oleic acid accumulation. The strong negative correlation between oleic and linoleic acids (r = −0.86) reflects a classical metabolic trade-off in seed oil biosynthesis. Higher oleic acid proportions observed in Aktobe populations may be associated with differences in local climatic conditions. However, the present dataset does not allow direct attribution of these differences to specific environmental drivers. This interpretation is supported by the PLS analysis, where mean annual temperature showed a negative association with oleic acid accumulation (Figure 12A). Similar temperature-dependent regulation of fatty acid desaturation has previously been reported in several oilseed crops [48].

Among all populations, AK01 demonstrated the most favorable nutritional profile, combining the highest total lipid content, elevated oleic acid concentration, and the best integrated TOPSIS ranking (Figure 13A). High-oleic oils are widely valued for oxidative stability and cardioprotective properties [50], making AK01 a promising accession for edible oil development and nutritional breeding. In contrast, KO01 accumulated the highest linoleic acid content and may therefore represent a valuable resource for developing PUFA-enriched germplasm adapted to arid environments. The Almaty populations accumulated relatively higher saturated fatty acids, including palmitic, stearic, and arachidic acids (Table 2). Although less desirable from a dietary perspective, these traits may be relevant for industrial applications requiring enhanced oxidative stability.

Taken together, the results demonstrate substantial intraspecific diversity in seed oil composition among *A. squarrosum* populations from Kazakhstan. Such diversity creates opportunities for targeted breeding strategies. For example, crosses between high-oleic populations, such as AK01, and PUFA-rich populations, such as KO01, could produce progeny with improved nutritional characteristics. These findings support the potential of *A. squarrosum* as a multifunctional pseudocereal suitable for domestication in climate-vulnerable dryland systems.

Although the present study identified several biologically relevant fatty acids and phenolic compounds, including linoleic acid, oleic acid, isorhamnetin, quercetin, and kaempferol, no direct biological activity assays were performed. Consequently, the reported biochemical profiles should be interpreted as indicators of potential functional value rather than evidence of biological activity. Future studies should combine metabolite profiling with antioxidant, anti-inflammatory, and other bioactivity assays to validate the functional significance of the identified populations.

### 4.3. Phenolic Composition and Potential for Medicinal and Functional Food Applications

The phenolic profiles identified in *A. squarrosum* populations indicate considerable nutraceutical and pharmaceutical potential. Isorhamnetin was consistently the dominant flavonoid across all populations (mean 0.42 ± 0.13 mg/g), followed by kaempferol and quercetin (Table 3), in agreement with previous species-wide studies [34]. These compounds possess well-documented antioxidant, anti-inflammatory, antimicrobial, and antitumor activities in previous experimental studies, suggesting that *A. squarrosum* may represent a promising source of metabolites with potential functional value [34]. However, direct assessment of biological activities was beyond the scope of the present study.

The predominance of isorhamnetin is noteworthy because previous studies have associated this flavonoid with cardiovascular protection, anti-inflammatory activity, and regulation of several signaling pathways, including PI3K/AKT, NF-κB, and MAPK [37]. The concentrations detected in this study, reaching up to 0.65 mg/g in Almaty populations, confirm that *A. squarrosum* represents a rich natural source of this compound (Table 3).

Populations from the Almaty region (AL01 and AL02) exhibited the highest accumulation of the entire phenolic compounds complex, including isorhamnetin, quercetin, kaempferol, rhamnetin, and tricin. The near-perfect positive correlations among these metabolites (r = 0.92–1.00) indicate highly coordinated biosynthetic regulation rather than independent accumulation (Figure 9). Such coordinated flavonoid enrichment likely reflects adaptive responses to environmentally heterogeneous and precipitation-rich habitats. Previous studies similarly demonstrated that the biosynthesis of phenolic compounds in *A. squarrosum* is strongly associated with adaptation to abiotic stress and regulated by extensive transcriptional networks [34].

The simultaneous elevation of quercetin and isorhamnetin may further enhance antioxidant efficiency because both compounds contribute to reactive oxygen species scavenging and lipid peroxidation inhibition [51]. Therefore, AL01 and AL02 may represent promising candidates for future nutraceutical development and for subsequent studies evaluating antioxidant-rich functional food ingredients. In contrast, the KO01 population exhibits elevated concentrations of coumarin, syringic acid, and vanillic acid, forming a distinct phenolic profile associated with adaptation to extreme heat and drought (Table 3). These metabolites are linked to antimicrobial defense, stress signaling, and oxidative stress mitigation [49], suggesting that KO01 relies on an alternative phenolic strategy compared with the flavonol-dominated Almaty populations.

Since the present study focused on leaf tissues, the results also highlight the importance of aerial biomass as a valuable source of bioactive compounds. Overall, the phenolic diversity observed across Kazakhstani populations demonstrates substantial potential for selecting elite accessions aimed at medicinal crop breeding, pharmaceutical applications, and functional food development.

### 4.4. Integrated Metabolic Relationships and Implications for Breeding Strategies

Correlation and network analyses revealed a modular organization of metabolism in *A. squarrosum*, providing important insights for breeding and domestication strategies. Two major metabolic modules were identified: a lipid-associated module composed of fatty acids and a phenolic-associated module dominated by flavonoids and phenolic acids (Figure 10). The relative independence of these modules reflects the distinct biochemical origins of lipid and phenylpropanoid biosynthesis and suggests partially competing carbon allocation pathways during seed and tissue development [52].

Weak but consistent negative correlations between total lipids and phenolic compounds (r = −0.06 to −0.26) support the existence of metabolic trade-offs between primary and secondary metabolism. Such trade-offs are common in stress-adapted plants, where limited carbon resources must be allocated between growth, storage compounds, and defensive metabolites [53]. These findings suggest that simultaneous optimization of lipid productivity and phenolic enrichment may require advanced breeding or genomic-assisted approaches rather than simple phenotypic selection.

Within the fatty acid network, stearic acid occupied a central topological position linking saturated and unsaturated fatty acid pathways. This result is biologically meaningful because stearoyl-acyl carrier protein desaturases regulate the conversion of stearic acid to oleic acid, thereby controlling downstream fatty acid desaturation [54]. The strong negative relationship between stearic and oleic acids (r = −0.79) confirms this regulatory role and identifies stearic acid as an important target for nutritional improvement. Consistent with this interpretation, stearic acid had a negative effect on the TOPSIS ranking, whereas total lipids and oleic acid had the strongest positive effects.

The phenolic module displayed extremely strong positive correlations among isorhamnetin, quercetin, kaempferol, rhamnetin, and tricin (r = 0.92–1.00), indicating coordinated biosynthetic regulation (Figure 9). This co-regulation is advantageous for breeding because selection for one marker flavonoid may simultaneously improve the entire antioxidant complex. Conversely, the strong negative relationship between the flavonoids cluster and coumarin-associated metabolites (r = −0.74 to −0.84) suggests alternative stress-adaptive phenolic strategies among ecotypes.

The integrated TOPSIS analysis, synthesizing all 16 biochemical traits, identified three complementary domestication trajectories. AK01 represents a nutritional ideotype optimized for high lipid and oleic acid accumulation; AL01 and AL02 represent medicinal ideotypes enriched in flavonoids; and KO01 represents a stress-resilient ideotype adapted to arid environments and enriched in PUFAs and defense-related phenolics. Integrating these ecotype-specific traits through structured crossing and genomic-assisted selection may provide an effective pathway toward the de novo domestication of *A. squarrosum* as a multifunctional crop for climate-resilient agriculture.

### 4.5. Prospects for De Novo Domestication and Climate-Resilient Crop Development

*A. squarrosum* represents a highly promising climate-resilient underutilized crop for future agriculture in arid and semi-arid environments. Our multi-population analysis reveals substantial biochemical diversity that aligns with ecological gradients, underscoring its potential as a multifunctional species combining drought tolerance, sandy-soil adaptation, high nutritional quality, and medicinal value.

Key findings highlight population-specific strengths. AK01 exhibited elevated total lipids (median near the upper end of the 8–14% range) and oleic acid, contributing to its top ranking in the TOPSIS analysis (driven primarily by total lipids at 48.6% and oleic acid at 25.5%). This aligns with its origin in the variable precipitation regime of western Kazakhstan. AL01 and AL02 stood out due to superior phenolic profiles, particularly high levels of isorhamnetin, kaempferol, quercetin, and rhamnetin, consistent with stronger flavonoid accumulation under heterogeneous southeastern conditions. These flavonoids correlate strongly (r = 0.92–1.00) and correspond to compounds previously reported to possess antioxidant and anti-inflammatory activities in experimental studies. KO01, from the southern arid zone, showed the highest linoleic acid and distinct phenolic patterns (elevated coumarin, syringic, and vanillic acids), potentially conferring advantages in extreme heat and aridity, as evidenced by its alignment with higher mean annual temperature in PLS biplots. Such ecotype differentiation mirrors patterns in Chinese populations, where drought stress modulates flavonoid pathways differently across precipitation gradients [35]. These results support ecotype-based domestication strategies, similar to successful de novo domestication efforts in other underutilized crops such as wild tomato and allotetraploid rice, in which targeted trait improvement preserved stress resilience while enhancing agronomic performance.

Future priorities should include genomic-assisted breeding to accelerate selection for yield and larger seed size, common garden trials to dissect genotype × environment interactions, and metabolomic-assisted domestication focusing on lipid and phenolic optimization. Comprehensive conservation of elite germplasm (AK01 for nutrition, AL01/AL02 for medicinal traits) is essential.

In conclusion, different populations of *A. squarrosum* are suited for distinct domestication goals, positioning the species as a valuable multifunctional crop candidate for sustainable agriculture under climate change. Although several identified metabolites, including isorhamnetin, quercetin, kaempferol, and unsaturated fatty acids, are known from previous studies to possess diverse biological activities, the present work did not include direct bioactivity assays. Therefore, the reported biochemical profiles should be interpreted as indicators of potential functional value, while experimental validation of antioxidant, anti-inflammatory, and other biological properties remains an important direction for future research.

### 4.6. Study Limitations

Several limitations should be considered when interpreting the present results. First, environmental association analyses were conducted using only five natural populations, which limits statistical power. Second, climatic associations were inferred from observational data rather than controlled experiments. Third, no direct biological activity assays were performed. Therefore, the reported correlations and functional implications should be interpreted as exploratory and should be validated in future studies.

## 5. Conclusions

This study demonstrated substantial biochemical diversity among natural populations of *A. squarrosum* from Kazakhstan, reflecting adaptation to contrasting arid environments. Linoleic acid was the dominant fatty acid across all populations, while isorhamnetin represented the major phenolic compound. Multivariate analyses revealed clear population differentiation based on lipid and phenolic profiles, with Aktobe populations characterized by elevated total lipid and oleic acid contents, whereas Almaty populations accumulated higher levels of flavonoids, including isorhamnetin, quercetin, and kaempferol. Future investigations should combine metabolite profiling with targeted functional assays to validate the biological relevance of the identified biochemical differences among populations. The obtained results confirm the high nutritional and bioactive potential of *A. squarrosum* and highlight its value as a multifunctional desert crop for future domestication. The identified ecotype-specific metabolic traits provide important genetic and biochemical resources for the development of functional foods, nutraceutical applications, and climate-resilient agricultural systems adapted to arid and semi-arid regions.

## Figures and Tables

**Table 1:** Characteristics of sampling sites.

Population	Region	Latitude	Longitude	Collection Date	Altitude	Soil Type
AK01	Aktobe	47°53′33.9″ N	59°46′26.33″ E	21 August 2025	191 m	Sandy soil
AK02	Aktobe	47°41′18.50″ N	60°17′42.29″ E	22 August 2025	261 m	Sandy soil
AL01	Almaty	44°43′17.94″ N	76°44′56.64″ E	3 September 2025	419 m	Sandy soil
AL02	Almaty	43°55′13.94″ N	77°15′46.01″ E	3 September 2025	528 m	Sandy soil
KO01	Kyzylorda	47°05′52.70″ N	63°15′44.22″ E	15 August 2025	94 m	Sandy soil

**Table 2:** Descriptive statistics of total lipid content, fatty acid composition, and phenolic compounds in *A. squarrosum* populations.

Traits	Mean ± SD	Min	Max	CV, %
Lipid and fatty acid traits				
Total lipids, %	9.69 ± 1.80	7.71	15.40	18.54
Arachidic acid, %	1.81 ± 0.47	1.07	2.60	25.81
Behenic acid, %	0.90 ± 0.18	0.59	1.15	19.61
Linoleic acid, %	54.35 ± 1.98	50.20	57.67	3.64
α-Linolenic acid, %	0.89 ± 0.32	0.23	1.52	35.58
Oleic acid, %	30.44 ± 4.70	24.80	40.10	15.44
Palmitic acid, %	9.47 ± 1.81	6.12	12.30	19.10
Stearic acid, %	2.20 ± 0.82	1.03	3.71	37.43
Phenolic compounds				
Coumarin, mg/g	0.03 ± 0.01	0.01	0.05	42.59
Isorhamnetin, mg/g	0.42 ± 0.13	0.24	0.65	31.63
Kaempferol, mg/g	0.18 ± 0.05	0.10	0.28	29.03
Quercetin, mg/g	0.14 ± 0.04	0.09	0.22	26.77
Rhamnetin, mg/g	0.07 ± 0.02	0.05	0.11	23.71
Tricin, mg/g	0.10 ± 0.02	0.06	0.14	22.63
Syringic acid, mg/g	0.06 ± 0.02	0.03	0.09	32.48
Vanillic acid, mg/g	0.09 ± 0.02	0.06	0.13	23.49

Note: Min—minimum, Max—maximum, SD—standard deviation, CV—coefficient of variation.

**Table 3:** Results of MANOVA for all traits, total lipids and fatty acids, and phenolic compounds among five populations of *A. squarrosum*.

**All Traits**						
	Df	Pillai’s Trace	Approx. F	Num Df	Den Df	*p* value
Population	4	3.385	11.351	64	132	<2.2 × 10^−16^
Residuals	45					
**Total Lipids and Fatty Acids**						
	Df	Pillai’s Trace	Approx. F	Num Df	Den Df	*p* value
Population	4	2.1971	6.2454	32	164	9.47 × 10^−16^
Residuals	45					
**Phenolic Compounds**						
	Df	Pillai’s Trace	Approx. F	Num Df	Den Df	*p* value
Population	4	2.8942	13.415	32	164	<2.2 × 10^−16^
Residuals	45					

Note: Df—degrees of freedom, Approx. F—Approximate F-statistic, Num Df—Numerator degrees of freedom, Den Df—Denominator degrees of freedom.

## Data Availability

The original contributions presented in this study are included in the article/Supplementary Materials. Further inquiries can be directed to the corresponding author(s).

## Supplementary Materials

The following supporting information can be downloaded at: https://www.mdpi.com/article/10.3390/biom16070950/s1, Figure S1: GC-MS total ion chromatogram (TIC) of the certified 37-component FAME standard mixture used for retention time verification and fatty acid identification; Table S1: Retention times of fatty acids and phenolic compounds identified in *A. squarrosum*; Table S2: Population-level means and standard deviations for total lipids, fatty acids, and phenolic compounds measured in five *A. squarrosum* populations; Table S3: Raw biochemical data of *A. squarrosum* populations from Kazakhstan; Table S4: TOPSIS scores and ranking of *A. squarrosum* samples based on integrated biochemical traits.

## Abbreviations

The following abbreviations are used in this manuscript:

GC-MS	Gas Chromatography–Mass Spectrometry
HPLC	High-Performance liquid chromatography
MANOVA	Multivariate analysis of variance
MAP	Mean annual precipitation
MAT	Mean annual temperature
PCA	Principal component analysis
PLS	Partial least squares
PUFA	Polyunsaturated fatty acid
TOPSIS	Technique for order preference by similarity to ideal solution
UPGMA	Unweighted Pair Group Method with Arithmetic Mean

## References

[1] Howden S.M., Soussana J., Tubiello F.N., Chhetri N., Dunlop M., Meinke H. (2007). Adapting agriculture to climate change. Proc. Natl. Acad. Sci. USA.

[2] Tilman D., Balzer C., Hill J., Befort B.L. (2011). Global food demand and sustainable intensification of agriculture. Proc. Natl. Acad. Sci. USA.

[3] Pirzadah T.B., Malik B. (2020). Pseudocereals as super foods of 21st century: Recent advancements. Curr. Res. Food Sci..

[4] Jacobsen S.E. (2006). The worldwide potential for quinoa (*Chenopodium quinoa* Willd.). Food Rev. Int..

[5] Chen G., Zhao J., Zhao X., Zhao P., Duan R., Nevo E., Ma X. (2014). A psammophyte *Agriophyllum squarrosum* (L.) Moq.: A potential food crop. Genet. Resour. Crop. Evol..

[6] Manoharan R., Asthana S., Somanathan Nair C., Gokhale T., Nishanth D., Jaleel A., Sood N. (2025). Exploring the hidden treasure in arid regions: Pseudocereals as sustainable, climate-resilient crops for food security. Front. Plant Sci..

[7] Li J. (2007). Flora of China. Harv. Pap. Bot..

[8] Fan S., Baskin C.C., Baskin J.M., Wang Y. (2017). The seed ecology of *Agriophyllum squarrosum*, a pioneer sand dune annual in Central Asia, with particular reference to seed germination. Seed Sci. Res..

[9] Genievskaya Y., Abugalieva S., Zhubanysheva A., Turuspekov Y. (2017). Morphological description and DNA barcoding study of sand rice (*Agriophyllum squarrosum*, Chenopodiaceae) collected in Kazakhstan. BMC Plant Biol..

[10] Li J., Qu H., Zhao H., Zhou R., Yun J., Pan C. (2015). Growth and physiological responses of *Agriophyllum squarrosum* to sand burial stress. J. Arid. Land.

[11] Chen J., Zhao X., Li Y., Luo Y., Zhang Y., Liu M., Li Y. (2021). Physiological responses of *Agriophyllum squarrosum* and *Setaria viridis* to drought and re-watering. Sci. Rep..

[12] Ma J., Liu Z. (2008). Spatiotemporal pattern of seed bank in the annual psammophyte *Agriophyllum squarrosum* Moq. (Chenopodiaceae) on the active sand dunes of northeastern Inner Mongolia, China. Plant Soil.

[13] Qian C., Yin H., Shi Y., Zhao J., Yin C., Luo W., Dong Z., Chen G., Yan X., Wang X.-R. (2016). Population dynamics of *Agriophyllum squarrosum*, a pioneer annual plant endemic to mobile sand dunes, in response to global climate change. Sci. Rep..

[14] Chen C., Zuo X., Zhao X. (2024). The effect of ecological characteristics on the domestication of sand rice (*Agriophyllum squarrosum*). PeerJ.

[15] Zhao P., Li X., Sun H., Zhao X., Wang X., Ran R., Zhao J., Wei Y., Liu X., Chen G. (2023). Healthy values and de novo domestication of sand rice (*Agriophyllum squarrosum*), a comparative view against *Chenopodium quinoa*. Crit. Rev. Food Sci. Nutr..

[16] Genievskaya Y., Karelova D., Abugalieva S., Zhao P., Chen G., Turuspekov Y. (2021). SSR-based evaluation of genetic diversity in populations of *Agriophyllum squarrosum* and *Agriophyllum minus*. Vavilov J. Genet. Breed..

[17] Jiao D., Liang Y., Zhou S., Wu X., Degen A.A., Hickford J., Zhou H., Cong H., Shi X., Ma X. (2022). Supplementing diets with *Agriophyllum squarrosum* reduced blood lipids, enhanced immunity and anti-inflammatory capacities, and mediated lipid metabolism in Tan lambs. Animals.

[18] Yang X., Fu W., Xiao L., Wei Z., Han L. (2024). Nutrition, health benefits, and processing of sand rice (*Agriophyllum squarrosum*): Comparisons with quinoa and buckwheat. Food Sci. Nutr..

[19] Jiecai Z., Pengshan Z., Xin Z., Xiaofei M., Yanli W., Qin Z., Guoxiong C. (2016). Biological characters, nutrient value and domestication feasibility of *Agriophyllum squarrosum*. J. Desert Res..

[20] Xu H.Y., Feng X.H., Zhang J.Y., Ma C.M. (2020). Recent progress in research on *Agriophyllum squarrosum* (L.) Moq. Shipin Kexue/Food Sci..

[21] Hai P., Li Q., Ma X.F., Jia H.Y., He Y.Q., Li X.Y., Luo Z.Q., Yang M.L., Gao Y., Wang H.P. (2025). Phytochemicals from a desert crop, sand rice (*Agriophyllum squarrosum*), and their inflammatory activity. ACS Omega.

[22] Winkel-Shirley B. (2002). Biosynthesis of flavonoids and effects of stress. Curr. Opin. Plant Biol..

[23] Hernández I., Alegre L., Van Breusegem F., Munné-Bosch S. (2009). How relevant are flavonoids as antioxidants in plants?. Trends Plant Sci..

[24] Shomali A., Das S., Arif N., Sarraf M., Zahra N., Yadav V., Aliniaeifard S., Chauhan D.K., Hasanuzzaman M. (2022). Diverse physiological roles of flavonoids in plant environmental stress responses and tolerance. Plants.

[25] Zhao P., Yan X., Qian C., Ma G., Fan X., Yin X., Liao Y., Fang T., Zhou S., Awuku I. (2024). Flavonoid synthesis pathway response to low-temperature stress in a desert medicinal plant, *Agriophyllum squarrosum* (sandrice). Genes.

[26] Patil J.R., Mhatre K.J., Yadav K., Yadav L.S., Srivastava S., Nikalje G.C. (2024). Flavonoids in plant-environment interactions and stress responses. Discov. Plants.

[27] Falcone Ferreyra M.L., Rius S.P., Casati P. (2012). Flavonoids: Biosynthesis, biological functions, and biotechnological applications. Front. Plant Sci..

[28] Nakabayashi R., Yonekura-Sakakibara K., Urano K., Suzuki M., Yamada Y., Nishizawa T., Matsuda F., Kojima M., Sakakibara H., Shinozaki K. (2014). Enhancement of oxidative and drought tolerance in *Arabidopsis* by overaccumulation of antioxidant flavonoids. Plant J..

[29] Vicente O., Boscaiu M. (2018). Flavonoids: Antioxidant compounds for plant defence… and for a healthy human diet. Not. Bot. Horti Agrobot. Cluj-Napoca.

[30] Shah A., Smith D.L. (2020). Flavonoids in agriculture: Chemistry and roles in biotic and abiotic stress responses, and microbial associations. Agronomy.

[31] Kawecki T.J., Ebert D. (2004). Conceptual issues in local adaptation. Ecol. Lett..

[32] Ferreyra M.L.F., Serra P., Casati P. (2021). Recent advances on the roles of flavonoids as plant protective molecules after UV and high light exposure. Physiol. Plant..

[33] Shi C., Liu H. (2021). How plants protect themselves from ultraviolet-B radiation stress. Plant Physiol..

[34] Zhou S., Yan X., Yang J., Qian C., Yin X., Fan X., Fang T., Gao Y., Chang Y., Liu W. (2021). Variations in flavonoid metabolites along altitudinal gradient in a desert medicinal plant *Agriophyllum squarrosum*. Front. Plant Sci..

[35] Zhao P., Yan X., Qian C., Ma G., Fang T., Yin X., Zhou S., Liao Y., Shi L., Fan X. (2025). Diversification of flavonoid accumulation among ecotypes of *Agriophyllum squarrosum* (L.) Moq. in response to drought stress. J. Arid. Land.

[36] Jaramillo S., Lopez S., Varela L.M., Rodriguez-Arcos R., Jimenez A., Abia R., Guillen R., Muriana F.J.G. (2010). The flavonol isorhamnetin exhibits cytotoxic effects on human colon cancer cells. J. Agric. Food Chem..

[37] Gong G., Guan Y.Y., Zhang Z.L., Rahman K., Wang S.J., Zhou S., Luan X., Zhang H. (2020). Isorhamnetin: A review of pharmacological effects. Biomed. Pharmacother..

[38] Rana J.N., Gul K., Mumtaz S. (2025). Isorhamnetin: Reviewing recent developments in anticancer mechanisms and nanoformulation-driven delivery. Int. J. Mol. Sci..

[39] (2017). Provides Official Guidelines for Near-Infrared Spectrometry in Cereals and Related Products.

[40] Ichihara K.I., Fukubayashi Y. (2010). Preparation of fatty acid methyl esters for gas-liquid chromatography. J. Lipid Res..

[41] Nallamuthu I., Devi A., Khanum F. (2020). Extraction processes with several solvents on total bioactive compounds in medicinal plants. Molecules.

[42] Sokal R.R., Michener C.D. (1958). A statistical method for evaluating systematic relationships. Univ. Kans. Sci. Bull..

[43] Hammer Ø., Harper D.A.T., Ryan P.D. (2001). PAST: Paleontological Statistics Software Package for Education and Data Analysis. Palaeontol. Electron..

[44] Pillai K.C.S. (1955). Some new test criteria in multivariate analysis. Ann. Math. Stat..

[45] Shannon C.E. (1948). A mathematical theory of communication. Bell Syst. Tech. J..

[46] R Core Team (2025). R: A Language and Environment for Statistical Computing.

[47] Schulte L.R., Ballard T., Samarakoon T., Yao L., Vadlani P., Staggenborg S., Rezac M. (2013). Increased growing temperature reduces content of polyunsaturated fatty acids in four oilseed crops. Ind. Crops Prod..

[48] Singh P., Arif Y., Bajguz A., Hayat S. (2021). The role of quercetin in plants. Plant Physiol. Biochem..

[49] Rao M.J., Zheng B. (2025). The role of polyphenols in abiotic stress tolerance and their antioxidant properties to scavenge reactive oxygen species and free radicals. Antioxidants.

[50] Huth P.J., Fulgoni V.L., Larson B.T. (2015). A systematic review of high-oleic vegetable oil substitutions for other fats and oils on cardiovascular disease risk factors: Implications for novel high-oleic soybean oils. Adv. Nutr..

[51] Zuo A., Yanying Y., Li J., Binbin X., Xiongying Y., Yan Q., Shuwen C. (2011). Study on the relation of structure and antioxidant activity of isorhamnetin, quercetin, phloretin, silybin and phloretin isonicotinyl hydrazone. Free Radic. Antioxid..

[52] Liang H., Ye F., Sun X., Wang C., Chen J., Xia T., Feng S., Wang Y., Jia C., Wu X. (2026). Analysis of metabolomics and transcriptomics of *Camellia drupifera* seeds during maturation provides new insights into bioactive compound biosynthesis. J. Future Foods.

[53] Caretto S., Linsalata V., Colella G., Mita G., Lattanzio V. (2015). Carbon fluxes between primary metabolism and phenolic pathway in plant tissues under stress. Int. J. Mol. Sci..

[54] Hernández M.L., Sicardo M.D., Alfonso M., Martínez-Rivas J.M. (2019). Transcriptional regulation of stearoyl-acyl carrier protein desaturase genes in response to abiotic stresses leads to changes in the unsaturated fatty acids composition of olive mesocarp. Front. Plant Sci..

