# Supplementary figures and images for: Integrated Analysis of Fatty Acids and Phenolic Compounds in Agriophyllum squarrosum (L.) Moq.: A Promising Desert Crop for Functional Foods and Sustainable Health
Source: Biomolecules. 2026 Jun 26;16(7):950. doi: 10.3390/biom16070950 (PMC13406645; doi:10.3390/biom16070950)

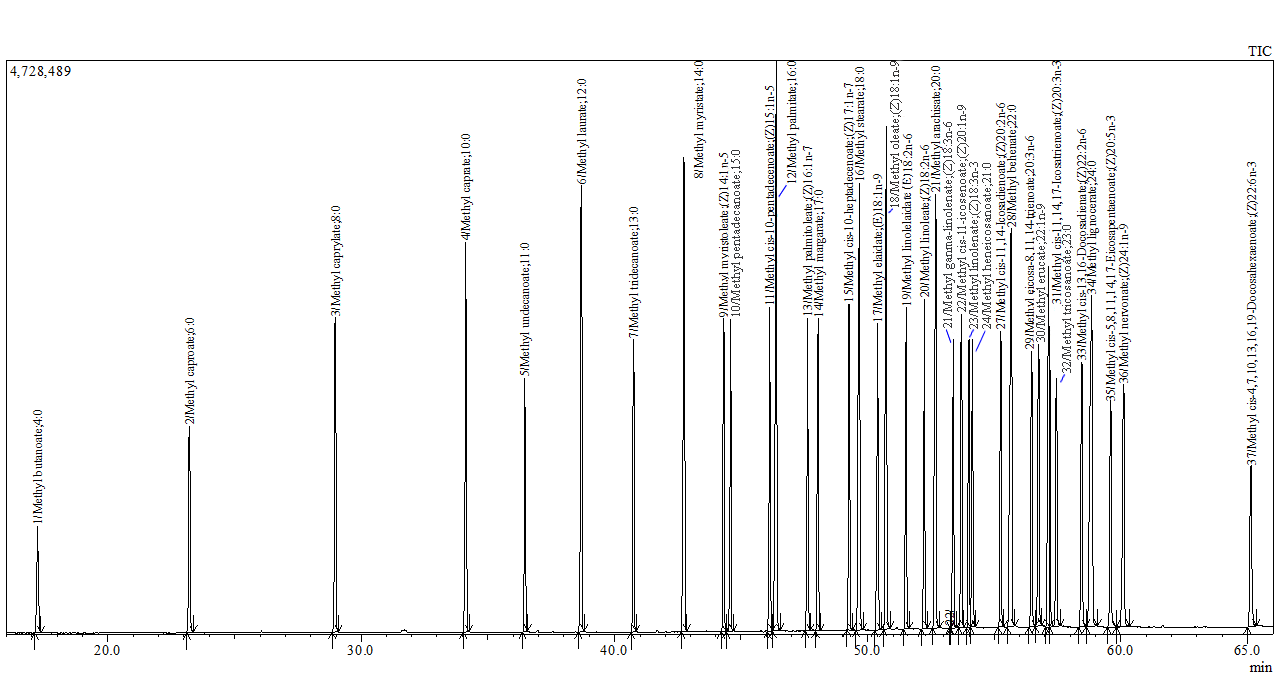

Supplement: Supplementary file 1 [file biomolecules-16-00950-s001.zip › Figure S1.png]
